# Anchorage and Stability of Orthodontic Mini Implants in Relation to Length and Types of Implants

**DOI:** 10.7759/cureus.73056

**Published:** 2024-11-05

**Authors:** Snehaa Selvaraj, Akshay Tandon, Deepak Chandrasekaran, Deenadayalan Purushothaman, Praveen Katepogu, Reshma Mohan, Nidhi Angrish

**Affiliations:** 1 Dental Surgery, Sri Ramaswamy Memorial (SRM) Kattankulathur Dental College and Hospital, Chennai, IND; 2 Orthodontics and Dentofacial Orthopedics, Sri Ramaswamy Memorial (SRM) Kattankulathur Dental College and Hospital, SRM Institute of Science and Technology, Chennai, IND; 3 Orthodontics and Dentofacial Orthopedics, Sri Ramaswamy Memorial (SRM) Kattankulathur Dental College and Hospital, Chennai, IND; 4 Orthodontics, Sri Ramaswamy Memorial (SRM) Kattankulathur Dental College and Hospital, SRM Institute of Science and Technology, Chennai, IND

**Keywords:** anchorage, implant lengths, mini implants, stability, types of implants

## Abstract

Anchorage control is crucial for achieving optimal results in orthodontic treatment. Scientific literature has documented the exploration of various methods to prevent anchorage loss, including the use of extraoral and intraoral devices. The advent of mini implants and micro implants has introduced new possibilities by allowing placement in previously inaccessible areas. These implants, also known as Temporary Anchorage Devices (TADs), significantly reduce the effort required to maintain anchorage. They are a reliable anchorage for orthodontic treatments because of their small size, ease of insertion and removal, and immediate force loading upon placement.

For individuals who are uncooperative or who have periodontal disease and alveolar bone loss, mini implants provide a stable substitute for anchorage during orthodontic therapy. Studies emphasize the importance of implant length and type in determining stability and success rates. Longer implants tend to increase stability, and self-drilling designs generally offer superior anchorage compared to self-tapping variants. Furthermore, titanium implants demonstrate higher success rates than stainless steel implants, highlighting their suitability for orthodontic applications.

## Introduction and background

For uncompromised results, anchoring control, either extra-oral anchorage or tooth-borne anchorage, is crucial during orthodontic therapy [1]. Using extra-oral anchorage is challenging, typically requires patient agreement, and can potentially be harmful. When the anchorage unit stays totally stable, it is said to be in "absolute anchorage." Orthodontic mini implants are used to create the absolute skeletal anchorage required for orthodontic treatment [2]. In orthodontics, traditional dental implants work well as anchorage devices. In recent years, the use of mini screw implants in orthodontics to improve anchorage has seen a significant advancement. In a few cases, these implants treat anterior tooth retraction, open bite correction, distalization, and tooth intrusion [3]. They provide a solid anchorage even under extreme circumstances and can be positioned in unusual locations, such as the alveolar bone of neighboring teeth, without causing damage to the roots or requiring time for osseointegration [4]. We refer to these implant-like devices as temporary anchorage devices (TADs) [5], also referred to as micro-implants, ortho-implants, or mini screw implants [2,6]. Mini screws' small size allows for placement between the roots of teeth, enhancing patient comfort [7].

Primary stability is the most critical factor in determining the success rate of mini implants in orthodontics. The mini implant's mechanical stability with the surrounding bone depends on a number of factors, such as the screw diameter, the operator's technique, and the amount and quality of cortical bone [3]. The bone's characteristics, the surgical method, and the implant's size and shape all have an impact on primary stability. The texture and form of the implant may also impact initial stability [8]. Although lengthening the screw does not increase its mechanical strength, it can successfully increase the initial stability of the mini implant [9]. Mini implants that are both short and have a modest diameter could compromise the primary stability [8]. When placing dental implants, another factor to consider is the activation of the molecular pathways involved in bone remodeling for osseointegration. These processes can initiate a series of inflammatory reactions by inducing the production of cytokines and chemokines, which contribute to the creation of a unique biochemical environment. Pro-inflammatory cytokines and osteoclastogenesis-related proteins largely cause peri-implantitis, one of the primary reasons for dental implant failure. Assessing these inflammatory mediators is crucial to improving the stability of orthodontic temporary anchorage devices [3].

Titanium or alloys make up the majority of orthodontic mini screws [10]. However, stainless steel mini implants are also frequently seen. Even though these two materials have different properties, they both meet the biomechanical requirements of orthodontic anchorage devices [11]. Observations show that stainless steel screws have a decreased bone interface compared to titanium screws. Because of their unique mechanical characteristics, which include excellent torsional resistance and flexural strength, stainless steel mini screws reduce the possibility of breaking during insertion [10]. Robert and other researchers worked with Branemark's discoveries in 1984, when they implanted titanium implants in rabbits. The study's findings indicate that titanium endosseous implants provide solid osseous anchorage. In order to stabilize invading upper anterior teeth, Creekmore employed Vitallium implants in 1988 [2]. Skeletal anchoring is now a more standardized and dependable method because of advancements in implant utilization and the creation of specialized, more modern systems. Orthodontists' appreciation of the effectiveness of implant-supported appliances may lead to a significant change in mechanotherapeutic techniques [1].

Patients with psychiatric illnesses (dysmorphia, psychoses), severe systemic disorders such as osteoporosis, blood disorders, alcoholism, drug abuse, heavy smoking, poor bone quality, or diabetes patients should not receive mini implants [2,12]. Mini implants have some drawbacks despite their therapeutic benefits, including the possibility of root injury, maxillary sinus or nasal floor perforation, dental ankylosis, and limited insertion space [4]. At this point, we think that implant loss can be caused by too much force on the mini implant, a large mucosa thick lever arm, peri-implantitis if the mucosa is left unconnected, not enough initial stability, and damage to the bone during insertion (overheating, compression) [13].

## Review

Methodology

To guarantee a thorough search, we utilized computerized techniques. Reputable websites like Google Scholar, PubMed, Science Direct, and Research Gate, published between 2006 and 2024, make up our search engines. We optimized our search by using a range of pertinent keywords, such as “mini implants,” “ortho implants,” “mini screws,” “implant lengths,” “types of implants,” “stability,” and “anchorage.” These were manually reviewed.

Mini implants

For many years, mini screws have served as an anchor during orthodontic force application and various forms of dental movement. According to the current study, there was a 90.2% success rate and a 9.3-fold increase in success likelihood compared to failure chance [7]. Orthodontic implants, made of alloplastic material, are surgically placed into or onto the jawbone. Mini implants consist of three parts: the head, neck, and body. The head functions as an abutment, the neck secures elastics, and the body embeds itself in the bone.

The biological properties of the mini implants should ensure efficient osseointegration, avoid injuring both rigid and soft tissues, contain no toxic diffuse material, and contain no substances that could trigger an allergic reaction. It shouldn't possess any possibility of cancer. It should be free of any flavor or smell, and its physical properties should include dimensional stability, sufficient resilience, strength, and the ability to withstand chewing or biting pressures [2]. Its size allows placement in any part of the alveolar bone, including the apical bone. In the maxilla, possible places for insertion are below the nasal spine, on the palate, at the alveolar process, on the infra zygomatic crest, or in the retromolar area. These are located at the mandible's symphysis [12,14]. An orthodontist or regular dentist should be able to conduct the surgical operation with ease and in a manner that allows for a quick recovery. The implant ought to be effortlessly detachable following orthodontic traction [1].

Biological responses and mechanical retention affect the stability of orthodontic mini implants [6]. Stability depends on two main factors: primary stability, which is achieved when the implant is first placed and mechanically bonds with the bone; and secondary stability, which is created as the bone continuously changes shape around the implant, making osseointegration easier. These components are vital for ensuring the effectiveness and longevity of mini implants in orthodontic treatment. Some important things that affect a mini implant's primary stability are where it is placed in relation to nearby roots, the shape and condition of the soft tissues around it, the technique used for placement, and the amount and length of orthodontic forces that are used [15]. The alveolar crest bone's distance from the mini implant influences its stability. Significant failure predictors include age, the mini implant site, and the distance to the alveolar crest [4].

Length

The length of mini implants, used as orthodontic anchoring, correlates with their success [16]. How long the mini implant is depends on the thickness and quality of the cortical bone, the angle at which it is inserted, the thickness of the transmucosal tissues, and the properties of other structures in the area [15]. When the cortical thickness is insufficient, we choose longer implants to ensure stability by interacting with the cancellous bone. Conversely, when cortical thickness provides sufficient stability, shorter implants are preferred. Wider and longer implants can compensate for insufficient primary stability in cases of low bone quality and quantity. Typically, experts recommend longer implants for the maxilla (8-10 mm) compared to the mandible (6-8 mm) [1,17,18]. The maxillary cortical surfaces are thinner and less dense than those of the mandible, necessitating the use of longer microimplants in the maxilla [1]. A comprehensive study found that mini implants, which have diameters between 1.4 and 1.9 mm and lengths between 5 and 8 mm, have the highest success rates (0.87, 95% confidence interval 0.80-0.92) [17].

The temporary anchorage devices should be at least 6 mm in length. In the event that 6 mm is taken as the minimum length and longer mini screw implants yield superior outcomes. According to Sarula et al., 8-mm-long temporary anchorage devices proved to be more successful than 6-mm-long temporary anchorage devices [19]. The bone screw, with a diameter of 1.5 mm, is designed for regions that support teeth, namely the space between teeth. The mandibular buccal shelf region, the midsagittal region of the hard palate, and the zygomatic buttress are examples of non-tooth-bearing locations that are intended for usage with the 2.0mm and 2.7mm diameter screws. The primary purpose of the 14mm and 17mm screw lengths is to insert them into the zygomatic buttress. You can use the bone height at the implant site to determine which of the three lengths (7, 10, and 12 mm) to use [1].

A systematic review conducted in 2010 that examined fourteen clinical trials revealed an 83.8 ± 7.4% overall mean success rate, with no discernible variations based on the patient's sex. The study revealed a lower success rate for mini screws with diameters between 1 and 1.1 mm compared to those with diameters between 1.5 and 2.3 mm. Avoid using mini screws with a diameter of 1.2 mm or less than 8 mm. Furthermore, a study found that the success rate of 6-mm mini screws was much lower than that of 8-mm ones (72% vs. 90%) [20]. A different study concluded that a diameter of 1.2 to 1.6 mm and a length of 6 to 7 mm are the ideal measurements for microimplants in the alveolar bone [21]. Long mini implants, in comparison to short mini implants, exhibit higher removal torque as well as a higher insertion torque requirement [8]. Increasing the length and placement height of mini implants, while decreasing their angulation, significantly increased the success rate [22].

Types

Cylindrical and Conical Type Mini Implants

The conical group exhibited high insertion torque, which may impact the healing of neighboring tissue, and high removal torque, which indicates strong initial stability [1]. Compared to the cylindrical, the conical had noticeably larger insertion torques (principal stabilities) [13] and greater removal torques [23].

Compared to cylindrical implants, conical-shaped mini implants provide tighter contact with the surrounding tissue, which contributes to their tendency to be more stable [6, 23]. This is because the conical implants' lower and upper sections have different sizes. The conical-shaped micro-implants may facilitate the mechanical retention between the implant and bone. Compared to a cylindrical mini implant, the taper-shaped one has 20%-30% less surface area. If the primary structure is stable enough, micromotion and bad tissue reactions may be less likely to happen during the loading and healing phases. For example, fibrous scar tissue may not form where the implant meets the bone. The use of conical implants has reduced implant failure [23]. The tapered-type screw took less time to penetrate the bone than the cylindrical form. [24] On the other hand, an excessive amount of insertion torque may cause the bone tissue to compress too much, which could weaken the mini implant's stability [23].

Titanium and Stainless Steel Type Mini Implants

Titanium, the most common material for mini implants, has better corrosion resistance, is more biocompatible than stainless steel (SS), and allows the surface of the mini implant to make direct contact with the patient's bone, aiding in the process of osseointegration. Stainless steel mini implants are also used due to their excellent mechanical qualities, higher resistance to breaking, and greater penetration capacity [11], as well as their affordability and ease of manufacturing [6]. The majority of research revealed that titanium and stainless steel had good success rates, ranging from 74.6% to 100%: 80.9%-100% [11].

The majority of the osseointegrating dental implants, orthodontic screws, and implants are made of titanium [2,6]. Medical applications utilize Grade I through IV titanium. Implant manufacturers frequently employ commercially pure titanium (C P Ti) due to its appropriate mechanical qualities and excellent biocompatibility. Because the screws were thinner, the use of titanium grades I through IV revealed failures in the production of mechanically retentive, non-osseointegrated mini screws. For orthodontic mini screws and mini implants, titanium alloy (Ti- 6Al-4V) (grade V) is the material of choice. The modulus of elasticity of titanium alloy (Ti-6Al-4V) is six times greater than that of bone, allowing for long and thin structures [2]. Titanium promotes improved osteointegration. Patients with titanium implants have higher levels of fibroblastic activity and lower levels of inflammatory cell responses compared to those with stainless steel implants [1]. Without expanding the MI diameter, the use of stainless steel mini implants can lower the risk of fracture [25].

We conducted a study comparing the stability of titanium and stainless steel mini screw implants with the same length and diameter during mass retraction of maxillary and mandibular anterior teeth. Every six months, the outcomes are measured in millimeters. The study found no discernible variation in the efficiency of retraction between mini screw implants made of titanium and stainless steel. With no statistically significant differences in the retraction outcomes, both types of implants demonstrated similar stability when it came to retracting the maxillary and mandibular anterior teeth [26]. The disadvantages of titanium mini implants include their higher price compared to stainless steel, as well as the requirement for pre-drilling in extremely dense bone [11]. Young's modulus of titanium and bone is the same as that of bone; a rise in Young's modulus and excessive bone stress will cause implant failure [1].

Self-Drilling and Self-Tapping

The self-tapping type inserts the micro implant into a pre-drilled tunnel in the bone, enabling it to tap as it drives in. Small-diameter microimplants typically use this technique, which indicates a prolonged treatment schedule [1,12,27]. The self-drilling type drives the micro implant directly into the bone, eliminating the need for pre-drilling. It is suitable for larger-diameter micro implants (greater than 1.5 mm) [1,12].

When it comes to anchorage, self-drilling micro implants are preferable to self-tapping micro implants. The peak insertion and removal torques are greater. Furthermore, self-drilling implants have a higher risk of breakage, but they also make better contact with the bone. They are especially advised for usage in the mandibular maxilla and regions with thin cortical bone. When comparing the self-drilling to the pre-drilling, the torque differences were greatest [22]. A study of the metal/osseous interface between drill-free screws and self-tapping screws shows that when drill-free screws are used, a lot more bone is left over at the screw thread location than when self-tapping screws are used [28]. With the self-drilling implants, there was more bone deposition in the apical region. Increasing the proportion of bone-implant contact will ultimately result in an increase in primary stability [29].

*Risk and Complications*

Mini implants can fail for a variety of reasons, including inadequate primary stability during placement, excessive forces applied during orthodontic treatment, or biological factors like peri implantitis [18]. The host factors include age, smoking, maintaining excellent dental hygiene, the implant site, keratinized tissue, cortical bone thickness, and bone density. The technical causes of failure include screw diameter, screw length, screw taper, screw thread shape, insertion technique, insertion torque, insertion angle, treatment duration, loading amount, loading direction, and microfracture of alveolar bone [30]. Mini implants fracture and bend during insertion and removal. Soft tissue irritation and inflammation can lead to peri implantitis, aphthous ulcers, and loosening of mini implants [2,18,27]. Place the implant in the interradicular bone between teeth; placing it in the root or periodontal ligament causes ankylosis or loss of vitality [18].

## Conclusions

Anchorage refers to the ability of mini implants to provide stability and resistance to orthodontic force movements. They are less expensive, allowing for early force application and shorter treatment times. Because of its size, it can be placed in any part of the alveolar bone, including the apical bone. Typically, orthodontic therapy ends with the temporary removal of mini implants. We can apply them to treat canted occlusal planes, open bites, and deep bites, among other conditions, such as incisor retraction, molar distalization, and anterior and posterior tooth intrusion. The stability of an implant immediately following insertion is known as primary stability; secondary stability results from bone remodeling.

The primary stability of orthodontic mini implants is significantly influenced by their length and type. The length of mini implants ranges from 6 mm to 12 mm. Longer implants can provide better anchorage in dense bone or when greater stability is required, whereas shorter implants may be used in areas with limited space or thinner bone. Compared to the cylindrical design, the conical thread design achieved greater primary stabilities. The most popular material for tiny implants in orthodontics is titanium. When it comes to anchorage, self-drilling micro implants are preferable to self-tapping micro implants. Successful use of mini implants often requires patient compliance with oral hygiene instructions; patients need to be told to practice daily plaque control at home and have professional assistance on occasion, just like with routine periodontal maintenance and avoiding habits that could compromise implant stability, such as chewing tough foods or using the implants as leverage.
